# Comparison of blood loss between tranexamic acid-soaked absorbable Gelfoam and topical retrograde injection via drainage catheter plus clamping in cervical laminoplasty surgery

**DOI:** 10.1186/s12891-022-05626-w

**Published:** 2022-07-14

**Authors:** Chong Chen, Yong-yu Ye, Yi-fan Chen, Xiao-xi Yang, Jin-qian Liang, Guo-yan Liang, Xiao-qing Zheng, Yun-bing Chang

**Affiliations:** 1 Department of Spine Surgery, Guangdong Provincial People’s Hospital, Guangdong Academy of Medical Sciences, No. 106, Zhongshan 2nd Road, 510080 Guangzhou, Guangdong China; 2 Department of Orthopedics, Peking University Third Hospital, Peking University, No. 49 Huayuan North Road, Haidian District, 100191 Beijing, China; 3 Department of Orthopedics, Peking Union Medical College Hospital, Chinese Academy of Medical Science, Peking Union Medical College, No.1 Shuaifuyuan Wangfujing, Dongcheng District, 100730 Beijing, China

**Keywords:** Tranexamic acid, Perioperative blood loss, Degenerative cervical myelopathy, Cervical laminoplasty

## Abstract

**Background:**

To compare the safety and efficacy of tranexamic acid (TXA)-soaked absorbable Gelfoam and the retrograde injection of TXA through a drain with drain-clamping in degenerative cervical laminoplasty patients.

**Methods:**

Patients were assigned into either TXA retrograde injection (TXA-RI), TXA-soaked absorbable Gelfoam (TXA-Gel), or control groups. The demographics, operative measurements, volume and length of drainage, length of hospital stay, complete blood cell count, coagulopathy, postoperative complications, and blood transfusion were recorded.

**Results:**

We enrolled 133 patients, with 44, 44, and 45 in the TXA-RI, TXA-Gel, and control groups, respectively. The baseline characteristics did not differ significantly among the three groups. The TXA-RI group exhibited a lower volume and length of postoperative drainage compared to the TXA-Gel and control groups (126.60 ± 31.27 vs. 156.60 ± 38.63 and 275.45 ± 75.27 mL; 49.45 ± 9.70 vs 58.70 ± 10.46 and 89.31 ± 8.50 hours, all *P* < 0.01). The TXA-RI group also had significantly shorter hospital stays compared to the control group (5.31 ± 1.18 vs 7.50 ± 1.25 days, *P* < 0.05) and higher hemoglobin and hematocrit levels (12.58 ± 1.67 vs 11.28 ± 1.76 g/dL; 36.62 ± 3.66% vs 33.82 ± 3.57%, both *P* < 0.05) at hospital discharge. In the TXA-RI and TXA-Gel groups, the D-dimmer (DD) and fibrinogen (FIB) were significantly lower than those in the control group after surgery (*P* < 0.05). None of the patients required blood transfusion. No complications, including thromboembolic events, were reported.

**Conclusion:**

Topical retrograde injection of TXA through a drain with drain-clamping at the conclusion of unilateral posterior cervical expansive open-door laminoplasty may effectively reduce postoperative blood loss and the length of hospital stays without increasing postoperative complications.

## Introduction

Cervical expansive open-door laminoplasty was first put forward in 1977 and was formally reported by Hirabayashi in 1983 [1]. With continuous improvements, it has become a commonly used technique for treating cervical spondylopathy combined with stenosis. Compared to the anterior approach, posterior expansive open-door laminoplasty surgery has a greater amount of bleeding, which could result in an increased risk of postoperative anemia, frequent requirements of allogeneic blood transfusion, longer bedtime, prolonged hospital stay, and more complications, such as infection and thrombotic events [2].

Tranexamic acid (TXA) is a synthetic lysine-analogue antifibrinolytic. TXA was patented in 1957, and has been used increasingly frequently in clinical practice [3]. Researchers have demonstrated that TXA can effectively reduce perioperative blood loss in trauma patients and patients undergoing joint arthroplasty [4, 5]. Nevertheless, due to the diversity in spinal surgical methods, the number of well-controlled trials that have been conducted is low.

TXA was recommended to be applied at the end of the degenerative lumbar scoliosis operation, at which point it is injected retrogradely through the drainage catheter. Then, the catheter is clamped for 1 h to ensure adequate contact time between the TXA and the wound to decrease the postoperative drainage. This method has been shown to reduce postoperative bleeding following lumbar scoliosis surgery [6].

However, to date there have been no reports on the effectiveness of the topical application of TXA in reducing post-operative bleeding following posterior expansive open-door laminoplasty. Here, we compared the efficacy of topical TXA injection (the method outlined above) and TXA-soaked absorbable Gelfoam to reduce post-operative blood loss and blood transfusion requirements in patients who underwent degenerative cervical laminoplasty surgery.

## Methods

### Study design and participants

This retrospective observation study enrolled patients who visited Guangdong Provincial People’s Hospital, met the selection criteria between July 2018 and July 2021. The study was approved by the Institutional Review Board (No. 2020191H).

The inclusion criteria were patients who: (i) had a definite diagnosis of degenerative cervical myelopathy (DCM) [7]; (ii) received unilateral posterior cervical expansive open-door laminoplasty from C3-C6/C7; and (iii) had complete medical records.

Patients who met the following criteria were excluded from the study: (i) patients with a history of cervical surgery; (ii) patients with anemia prior to the operation (male hemoglobin < 13 g/dL, female hemoglobin < 12 g/dL); (iii) patients with an allergy to TXA; (iv) patients exhibiting any type of coagulopathy; (v) patients undergoing anticoagulant and/or antiplatelet treatment; (vi) patients with a previous history of thromboembolic events, including deep vein thrombosis (DVT), ischemic heart disease, pulmonary embolism (PE), transient ischemic attack, stroke, or sub-arachnoid hemorrhage; and (vii) patients with chronic liver disease, renal insufficiency (creatinine > 2.0 mg/dL), or pregnancy.

#### Surgical procedure

The patients were given general anesthesia and set in a prone position on the operating table. The nuchal fascia was longitudinally divided in line with the midline skin incision, exposing the posterior surfaces of the laminae between C3 and C6/C7 and the medial border of the facet joints. Next, the paravertebral muscles were dissected from the spinous processes of C3–C6/C7 [8].

The right lamina was usually selected as the hinge side, and the left side was the conventional open-door side. A high-speed matchstick burr was used to create a gutter by drilling through the inner cortex at the medial border of the facet joints, which served as hinges, while a second gutter on the contralateral side penetrated the inner cortex and the spinal canal as the open side. Using the hinge-side as a fulcrum, the vertebral plate was slowly and gently turned to the hinge side [9]. A periosteal detacher was then utilized to conduct the auxiliary distraction of the opening side along the inner edge of the lateral mass in order to attain an appropriate opening distance. Suitably-sized titanium plates were fixed separately on the lateral mass and vertebral plate [10]. Next, the surgeon adjusted the position of the titanium plates and fixed them in place using screws, and the final position was confirmed through C-arm fluoroscopy.

Intravenous fluid infusions to all patients consisted of a 500 mL hydroxyethyl starch injection or succinylated gelatin injection plus a 500 mL sodium lactate Ringer’s injection. All patients exhibiting a mean arterial blood pressure (MAP) of approximately 20% below the preoperative value, and with a minimum MAP of 60 mmHg, were treated to control hypotension. Controlled hypotension was maintained until the procedure had been nearly completed, and then the MAP was returned to the baseline pressure so that the surgeon could determine whether there was bleeding.

#### Group assignment and interventions

Participants were assigned into three groups: the TXA retrograde injection (TXA-RI), TXA-soaked absorbable Gelfoam (TXA-Gel), and control groups, based on the clinical interventions received. The control group was not administered TXA via Gelfoam or catheter.

**Fig. 1:**
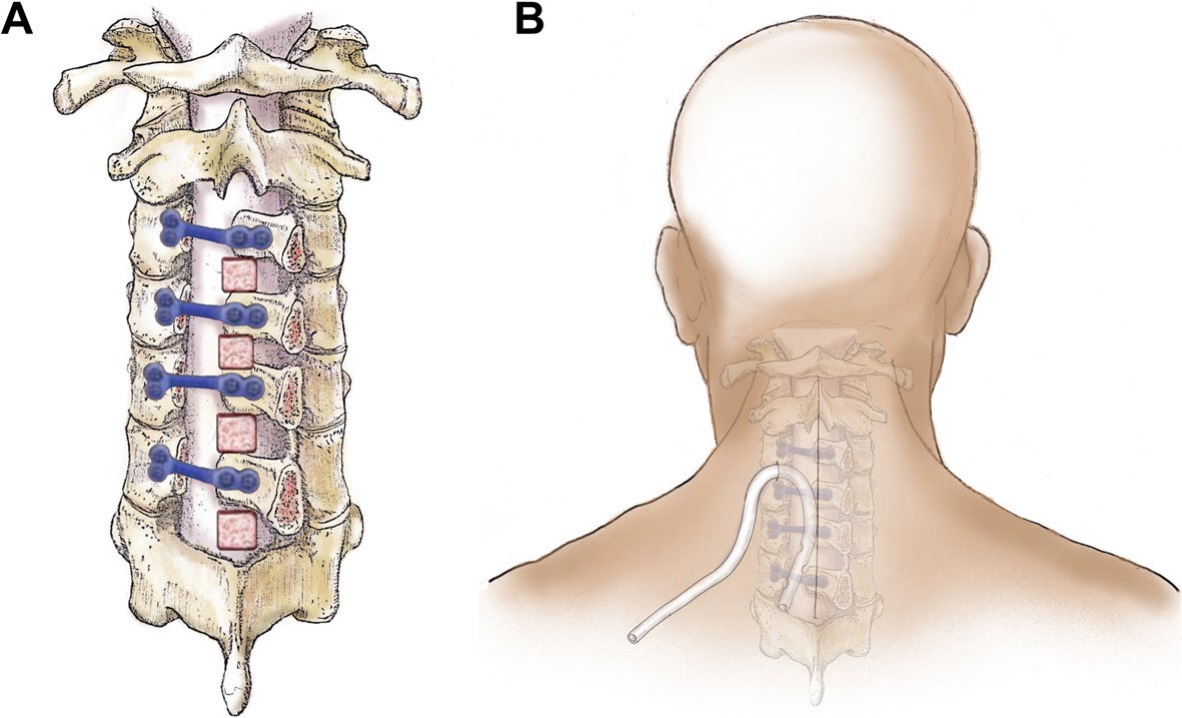
Schematic diagram of the topical application of tranexamic acid-soaked absorbable Gelfoam (**A**) and retrograde injection via drainage catheter (**B**)

In the TXA-Gel group, after adequate hemostasis had been achieved, according to the size of the exposed spinal dura, the Gelfoam (Kuai Kang, China) soaked with TXA (Pude Pharma, China) (1 g in 20 mL saline solution) was cut into a proper shape to ensure that the entire dura was covered before stitching the wound (Fig. 1A).

For the TXA-RI group, a deep drainage catheter was placed below the fascia at the conclusion of the operation. This step was followed by the retrograde injection of TXA via a catheter. Twenty milliliters of saline containing 1 g of TXA was injected into the wound. The catheter was clamped for 1 h (Fig, 1B).

The amount of drainage in the Hemovac (Leadgem Medical, China) was measured and recorded every 8 h. The catheter was removed when the drainage pull out was less than 20 mL per 8 hours or 60 mL per 24 hours.

#### Data collection

The demographic data, operative measurements, volume and length of drainage, blood transfusion requirements, length of hospital stay, laboratory results (complete blood cell count and coagulation panel), postoperative complications, and blood transfusion requirements were recorded.

The levels of hemoglobin and coagulation function were measured pre-operatively, on postoperative days (POD) 1 and 3, and on the day of hospital discharge.

The total patient’s blood volume (PBV) was calculated based on the following formula as described by Nadler et al. [11]: PBV (L) = *k*1 × height (m)^3^ + *k*2 × weight (kg) + *k*3. For male patients, *k*1 = 0.3669, *k*2 = 0.03219, and *k*3 = 0.6041, and for female patients, *k*1 = 0.3561, *k*2 = 0.03308, and *k*3 = 0.1833.

The Gross formula was used to calculate the total blood loss (TBL) as follows [12]: TBL = PBV × (Hct_pre_ – Hct_post_)/Hct_ave_, where Hct_pre_ was the preoperative hematocrit (Hct) level, Hct_post_ was the Hct level on postoperative day 3 or the day of discharge (whichever was lower), and Hct_ave_ was the average of the Hct_pre_ and Hct_post_ values.

The possibility of DVT and/or PE was observed for 4 weeks after the surgery. The wound healing conditions, such as skin necrosis, hematoma, and infection, were also monitored.

#### Statistical analysis

The categorical data are shown as number and percentage, while the continuous data are shown as the mean and standard deviation. The differences in the perioperative data among the three groups were analyzed by one-way ANOVA, Pearson χ^2^-test, or Fisher’s exact test with Bonferroni adjustment, when appropriate. All statistical analyses were conducted using SPSS software version 22.0 (IBM, NY, USA). Statistical significance was considered to be *P < 0.05*.

## Results

**Fig. 2:**
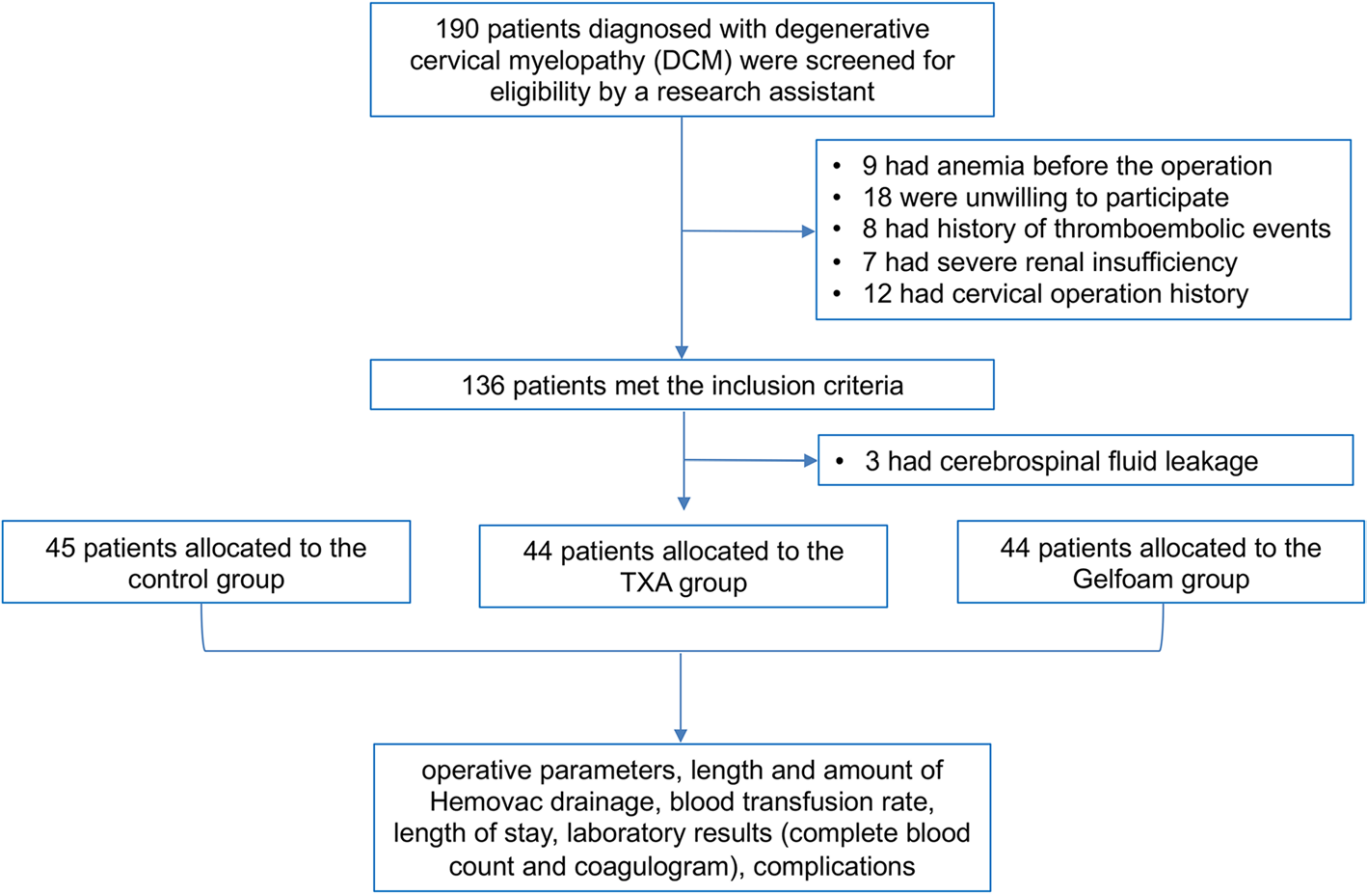
Patient selection flowchart

This retrospective, nonrandomized, case-control study included 133 patients, with 44, 44, and 45 patients in the TXA-RI, TXA-Gel, and control groups, respectively. The selection process is shown in Fig. 2. There was no statistical difference in the baseline characteristics among the three groups (Table 1).

**Table 1:** Comparisons of the baseline characteristics among different groups

	Control group (*n* = 45)	TXA-Gel group (*n* = 44)	TXA-RI group (*n* = 44)	*P*
Age (years) [range]	65.12 ± 8.72	64.43 ± 8.68	63.82 ± 9.43	>0.05
Sex (female/male)	9/36	11/33	9/35	>0.05
BMI [range]	21.37 ± 2.27	22.14 ± 1.90	21.44 ± 2.18	>0.05
Etiology of DCM (n)				
Cervical myelopathy	31	32	33	>0.05
OPLL	14	12	11	>0.05
Comorbidity	2	3	3	>0.05
Surgical level				
C3–6	39	39	38	>0.05
C3–7	6	5	6	>0.05

*BMI* Body mass index, *DCM* Degenerative cervical myelopathy, *OPLL* Ossification of the posterior longitudinal ligament

As shown in Table 2, the volume of the postoperative drainage was smaller in the TXA-RI group than in the TXA-Gel and control groups (126.60 ± 31.27 vs. 156.60 ± 38.63 and 275.45 ± 75.27 mL, both *P* < 0.05). It is worth noting that on the first day after surgery, the drainage was lower in the TXA-RI and TXA-Gel groups than in the control group (*P* < 0.05). The drainage of the TXA-RI group was the lowest in the first 8-h period (*P* < 0.05). Although there were no statistically significant differences in the duration of surgery and estimated blood loss among the three groups, the total blood loss was lower in the TXA-RI and TXA-Gel groups than in the control group (251.80 ± 54.26 vs. 417.20 ± 89.14, 295.73 ± 68.54 vs. 417.20 ± 89.14 mL, *P* < 0.05, respectively). The mean hematocrit and hemoglobin levels were significantly higher in the TXA-RI group compared to those in the control group at the time of discharge (12.58 ± 1.67 g/dL vs 11.28 ± 1.76 g/dL; 36.62 ± 3.66% vs 33.82 ± 3.57%, *P* < 0.05, respectively) (Table 2).

**Table 2:** Outcome comparisons among three groups

	Control group (*n* = 45)	Gelfoam group (*n* = 44)	TXA group (*n* = 44)
Op time (min)	91.20 ± 18.52	89.80 ± 19.73	87.20 ± 21.69
EBL (mL)	125.20 ± 45.44	138.60 ± 52.76	123.30 ± 35.83
Hemovac drainage (mL)			
The first 8 h postoperatively	65.17 ± 17.66	37.72 ± 14.54^†^	23.47 ± 10.66^*, ‡^
The second 8 h postoperatively	53.79 ± 19.88	21.44 ± 12.31^†^	20.53 ± 13.28^*^
The third 8 h postoperatively	33.64 ± 14.74	15.52 ± 13.54^†^	13.28 ± 4.54^*^
Total drainage	275.45 ± 75.27	156.60 ± 38.63^†^	126.60 ± 31.27^*, ‡^
Total blood loss	417.20 ± 89.14	295.73 ± 68.54^†^	251.80 ± 54.26^*^
Length of drainage (h)	89.31 ± 8.50	58.70 ± 10.46^†^	49.45 ± 9.70^*, ‡^
Hemoglobin (g/dL)			
Baseline	13.53 ± 0.98	13.45 ± 1.23	13.22 ± 1.17
Day 1 postoperation	11.81 ± 1.54	12.49 ± 1.42^†^	12.68 ± 1.64^*^
Day 3 postoperation	11.18 ± 1.97	11.81 ± 1.36	12.14 ± 1.62^*, ‡^
At discharge	11.28 ± 1.76	12.14 ± 1.53^†^	12.58 ± 1.67^*, ‡^
Hematocrit (%)			
Baseline	40.10 ± 4.37	39.16 ± 3.74	41.24 ± 3.17
Day 1 postoperation	33.76 ± 4.28	34.12 ± 3.82	37.12 ± 3.94^*^
Day 3 postoperation	32.52 ± 3.78	34.75 ± 3.56^†^	35.95 ± 4.34^*^
At discharge	33.82 ± 3.57	35.88 ± 4.12^†^	36.13 ± 3.87^*^
Postoperative length of stay (d)	7.50 ± 1.25	5.64 ± 0.96^†^	5.31 ± 1.18^*^

*EBL* Estimated intraoperative blood loss, *Op time* Operation time

* *P* < 0.05 between TXA group and Control group; † *P* < 0.05 between Gelfoam group and control group; ‡ *P* < 0.05 between TXA group and Gelfoam group

In the TXA-RI group, the drainage duration was significantly shorter than those in the TXA-Gel and control groups (49.45 ± 9.70 vs 58.70 ± 10.46, 49.45 ± 9.70 vs 89.31 ± 8.50 hours, *P* < 0.01, respectively). The TXA-RI and TXA-Gel group also had a significantly shorter hospital stay than the control group (5.31 ± 1.18 and 5.64 ± 0.96 vs 7.50 ± 1.25 days, *P* < 0.05, respectively) (Table 2).

**Fig. 3:**
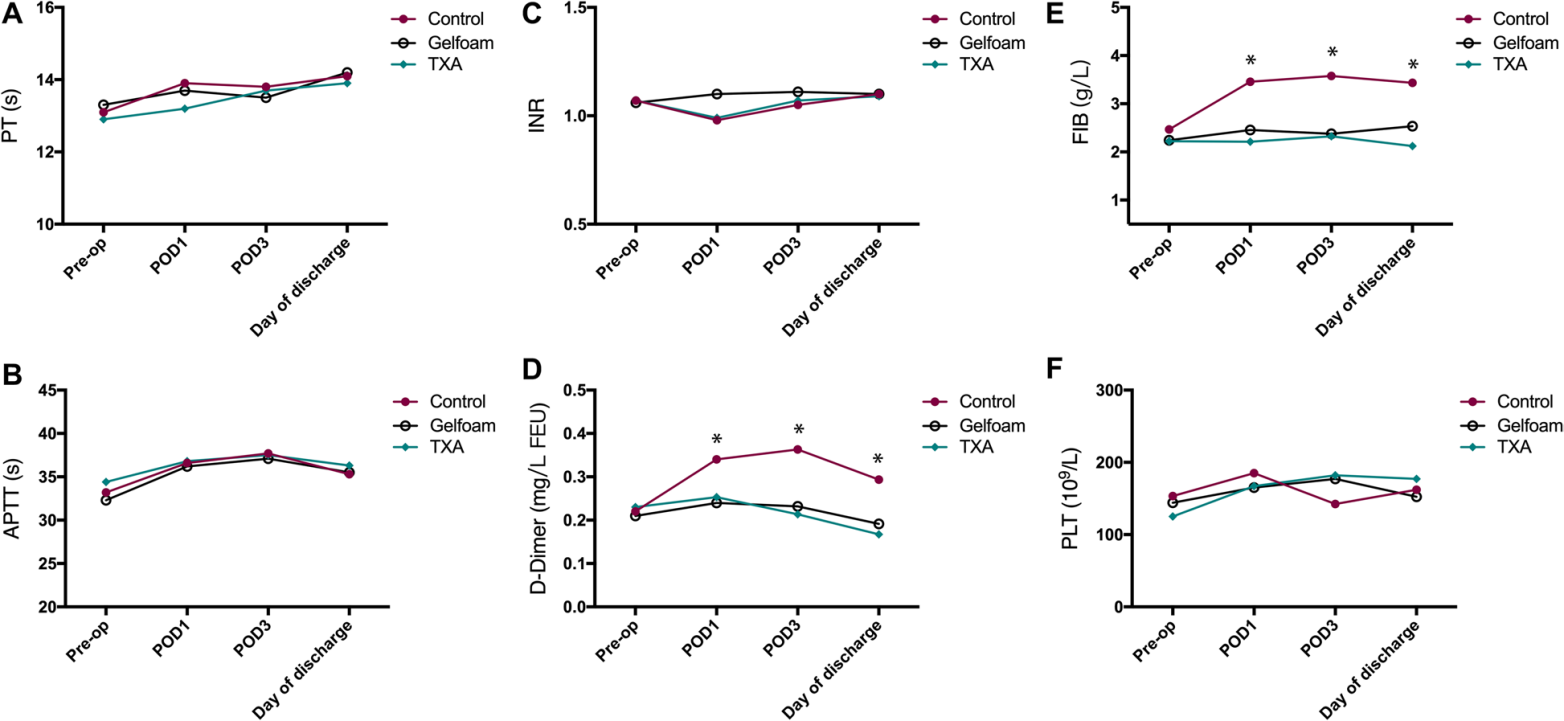
Perioperative coagulation panels in the three study groups. No significant differences were detected among the three groups for the prothrombin time (PT), activated partial thromboplastin time (APTT), international normalized ratio (INR) or blood platelet count (PLT) on four perioperative time points, but in the TXA-RI and TXA-Gel groups, the D-dimmer (DD) and fibrinogen (FIB) were significantly lower than those in the control group after surgery. * *P* < 0.05

Regarding the perioperative coagulation panel and blood platelet count (PLT), no significant differences were detected among the three groups for the prothrombin time (PT), activated partial thromboplastin time (APTT), international normalized ratio (INR) or PLT on four perioperative time points (*P* > 0.05), but in the TXA-RI and TXA-Gel groups, the D-dimmer (DD) and fibrinogen (FIB) were significantly lower than those in the control group after surgery (*P* < 0.05) (Fig. 3).

No patients required blood transfusions during or following surgery. No group experienced thromboembolic events or complications.

## Discussion

The abundant soft tissue in the dorsal side of the neck can increase the risk of bleeding during incision and dissection [13]. For laminoplasty, the risk of bleeding also increases with a large cancellous bone exposure and a long surgical duration compared with anteiror cervical surgery. In cases of developmental cervical spinal stenosis, the ossification of the posterior longitudinal ligament (OPLL) and the high pressure of the venous plexus in the intraspinal canals can further increase the risk of bleeding after the cervical spinal canal is enlarged.

TXA is a synthetic lysine-analogue antifibrinolytic that competitively inhibits the activation of plasminogen to plasmin. In addition, high concentrations of TXA non-competitively blocked plasmin. In fact, TXA can inhibit fibrin clot dissolution and degradation due to plasmin [14]. Tsutsumimoto et al. [15] found that intravenous injection of TXA in cervical laminoplasty reduced postoperative blood loss, which was confirmed by Yu et al. [16]. Moreover, multiple studies have indicated that TXA can reduce intraoperative blood loss and transfusion for scoliosis surgery [17–20].

TXA-soaked Gelfoam sponge applied locally is a safe, effective method that can be used to reduce post-operative blood loss and blood transfusions among low-risk adult lumbar spine surgery patients [21]. In addition, topical use of TXA before wound closure has been shown to decrease blood loss in patients undergoing posterior lumbar spinal fusions [22].

Both collagen hemostatic sponge and topical TXA were effective and safe in patients with lumbar degenerative diseases, with TXA showing a better efficacy [23]. Another study demonstrated that topical injection of TXA retrogradely via a drainage catheter, followed by clamping the catheter for 1 h, effectively decreased both postoperative blood loss and the length of hospital stay without increasing complications in patients with degenerative lumbar scoliosis [6]. Previous studies also demonstrated that local and intravenous use of low-dose TXA could enhance the hemostatic effect [24, 25], sugesting a high dose of TXA might not be required to achieve hemostasis.

Recently, the results of a prospective, double-blinded, randomized, controlled trial suggested that a multiple-dose regimen of TXA, either through oral or intravenous application, could inhibit the postoperative inflammatory response, and was a safe and effective method to control the postoperative blood loss and decrease the postoperative transfusion requirements in patients with adolescent idiopathic scoliosis (AIS) who underwent scoliosis surgery [26].

Wang et al. [27] reported that TXA treatment in thoracic spinal interbody fusion resulted in the reduction of both visible and hidden blood loss without causing prethrombotic molecular markers. Whereas, some studies have reported that the use of TXA does not significantly reduce intraoperative bleeding [28]. Because intravenous used TXA reaches a peak blood concentration approximately 1 h after infusion and keep the duration of action is 3 hours, with a mean half-life of 0.68 hours [28–30]. According to this pharmacological characteristic, the best hemostatic effect can be achieved by administering 1 g of TXA 30 min before the operation.

Bleeding during the operation, however, comes mainly from fresh blood vessel bleeding, and as a result, it will take some time from bleeding to platelet and fibrin aggregation to form thrombosis and hemostasis, and, consequently, to observe the antifibrinolytic effect of TXA. In other words, the peak plasma concentrations of intravenously-administered TXA and its beneficial effects are achieved at the end stage of surgery.

At present, it only takes approximately 90 min to perform the laminoplasty procedure. Therefore, at the time when the TXA starts to be effective, the operation has gone through the steps that lead to bleeding, which cannot reflect the effect of significantly reducing bleeding. For the topical usage of TXA, the peak concentration is reached 30 min after application. With a half-life of 1.26 hours, topical TXA has a longer half-life than intravenously-administered TXA [29]. In our study, significant differences were observed in the postoperative drainage on the first day, especially the first 8 h after the operation. There was no significant difference in the estimated intraoperative blood loss among the three groups.

Moreover, no thromboembolic events or complications occurred in our groups. As illustrated in one meta-analysis, the topical application of TXA during spinal surgery decreased the total blood loss and drainage volume and allowed patients to maintain a higher postoperative hemoglobin level without increasing the risk of infection, hematoma, DVT, and PE [31]. In fact, antifibrinolytic drugs, such as TXA, stop bleeding by slowing down the degradation of blood clots, rather than by changing the coagulation function of patients. The use of TXA during an operation can reduce the concentration of D-dimers, but has no significant effect on the coagulation system and does not promote thrombosis formation.

PT, APTT, INR, FIB, DD and PLT levels were used to assess the perioperative coagulation functions. The levels of DD and FIB were monitored during the spinal surgery, and suggested that TXA impeded the fibrinolytic pathway through reducing the consumption of fibrinogen and clot dissolution, as indicated by the decreased formation of DD [32]. Our results showed that there was no statistically significant differences among the three groups for majority parameters. But in the TXA-RI and TXA-Gel groups, the DD and FIB were significantly lower than those in the control group after surgery, which was consistent with studies.

Sharma et al. [33] reported that the use of TXA was a strong independent predictor of postoperative generalized seizures, and that TXA treatment caused a 2.5-fold increase in mortality rate. Studies in animals have also shown that the topical administration of TXA to the central nervous system may cause seizures in a dose-related fashion [34, 35]. Accidental intrathecal injection of TXA was reported to cause seizures in humans [36, 37]. The possible mechanism might be due to the binding of TXA to the GABAA receptors, with subsequent blockage of = GABAA-mediated inhibition in the central nervous system [38]. Lecker et al. [39] reported that TXA was structurally similar to glycine, and showed that TXA competitively inhibited the glycine inhibitory receptors in cortical and spinal cord neurons in a rat model. Thus, in our study, we excluded topical usage of TXA for patients with cerebrospinal fluid leakage to avoid side effects in the central nervous system.

In the present study, none of the patients required a blood transfusion. Other studies have shown clear benefits in terms of mortality, morbidiy, and medical expenses due to TXA treatment. One meta-analysis reviewed more than 100 clinical trials comparing TXA treatment to no TXA treatment or a placebo in over 10,000 surgical patients. The results found overwhelming evidence that TXA reduced blood transfusions by 38% [40]. Furthermore, the reduction in hospital stay by almost 2 days in the TXA treatment group may also correlate with less medical expenses for patients.

## Limitations

The limitations of our study included retrospective design, single-center research, and a small number of patients. Future prospective clinical trials are required to confirm our study results.

## Conclusions

In degenerative cervical laminoplasty surgery, the retrograde topical application of TXA through a drainage catheter, with the drain clamped for 1 hour at the conclusion of the surgery, can effectively decrease postoperative blood loss and the length of hospital stays without increasing postoperative complications.

## Data Availability

The datasets generated and analysed during the current study are not publicly available due privacy policy from our institution, but are available from the corresponding author on reasonable request.
